# Longitudinal Profiles of Psychological Well-Being and Health: Findings From Japan

**DOI:** 10.3389/fpsyg.2019.02746

**Published:** 2019-12-10

**Authors:** Jiah Yoo, Carol D. Ryff

**Affiliations:** ^1^Department of Psychology, University of Arizona, Tucson, AZ, United States; ^2^Department of Psychology, University of Wisconsin–Madison, Madison, WI, United States

**Keywords:** well-being, longitudinal, culture, protective factors, physical symptoms, chronic illness, healthy functioning

## Abstract

Studies have reported relationships between psychological well-being and physical health in Western cultural contexts. However, longitudinal associations between well-being and health have not been examined in other cultures where different values and beliefs about well-being exist. This paper examined whether longitudinal profiles of well-being predict prospective health among Japanese adults. Data came from 654 people who completed two waves of the Midlife in Japan (MIDJA) Study collected 4–5 years apart. Health outcomes were assessed with subjective health, chronic conditions, physical symptoms, and functional health. The results showed that persistently high well-being predicted better health over time. High-arousal positive affect, which is relatively less valued in Japanese culture, was also associated with better health. The findings add cross-cultural evidence to the cross-time link between well-being and health.

## Introduction

Psychological well-being is multifaceted including both eudaimonic (e.g., purpose, fulfillment) and hedonic (e.g., feeling good) aspects. Psychological well-being has been shown to predict a wide array of health outcomes such as better subjective health (Benyamini et al., 2000), fewer chronic conditions (Friedman and Ryff, 2012), and lower rates of mortality (Boyle et al., 2009; Diener and Chan, 2011). Most prior findings have, however, been based on cross-sectional data, making the causal nature of the relationships unclear. The limited number of studies using longitudinal data have shown that higher levels of purpose in life, a component of psychological well-being, predicts future health outcomes, after controlling for baseline health (e.g., Boyle et al., 2009; Kim et al., 2013). In addition, cumulative profiles of well-being focused on comparisons between persistently high or low levels of well-being predict better cross-time health, measured in terms of subjective health, chronic conditions, and functional impairment (Ryff et al., 2015).

Notably missing in this literature is research from non-Western cultural contexts. This omission is important because psychological well-being is known to be shaped by cultural values and beliefs (Kitayama et al., 2000; Kitayama and Park, 2007; Fulmer et al., 2010; Karasawa et al., 2011; Ryff et al., 2014; Miyamoto et al., 2019). Thus, it is unknown whether distinct aspects of well-being benefit health in different cultural contexts. To address this issue, the current study examined longitudinal links between diverse indicators of well-being and self-reported health in Japan, using assessments comparable to a prior study in the United States (Ryff et al., 2015; Radler et al., 2018). Japan is a relevant comparison because it is comparable to advanced Western countries in modern lifestyles and economic development, but also encompasses substantial differences in conceptions of self and relationships with others (Markus and Kitayama, 1991).

Although some dimensions of well-being have been considered important in both Japan and Western countries, other dimensions are thought to be particularly important in Western cultural contexts but less relevant to Japanese (Kitayama et al., 2000; Tsai, 2017). For example, there are notable parallels between purpose in life, an aspect of well-being in the West that has increasingly been linked to positive health outcomes (e.g., Boyle et al., 2009; Kim et al., 2013; Hill and Turiano, 2014) and the Japanese concept of *ikigai*, which refers to what makes life worth living. *Ikigai* is believed to be the most common indicator of psychological well-being in Japanese culture (Nakanishi, 1999). Kumano’s (2017) content analysis of how Japanese define *ikigai* in everyday language further showed that the concept comprises a sense of accomplishment, devotion (despite difficulties), and social and benevolent contribution. These elaborated ideas suggest possible overlap with other aspects of well-being such as personal growth and positive relations with others^1^. Pertinent to health, *ikigai* has repeatedly predicted better health in Japan, including lower mortality and incidence of functional disability (Sone et al., 2008; Tanno et al., 2009) and greater physical activity (Ueshima et al., 2010).

In contrast, other aspects of well-being have shown differences in cultural values and meanings. Two examples include autonomy and positive affect, particularly high-arousal positive affect (e.g., excited, enthusiastic). Individuals in Western cultures tend to experience self as an independent entity from their social surroundings, whereas self-concept in East Asian cultures is defined via one’s interdependent relations to other people (Markus and Kitayama, 1991). Within the interdependent conception of self, attuning and adjusting to others is crucial to social functioning and individual well-being (Kitayama and Markus, 2000). Thus, autonomy, defined as a commitment to following individual convictions and beliefs may be contrary to virtuous living in Japanese compared to Western cultural contexts (Oishi, 2000; Karasawa et al., 2011). Similarly, high-arousal positive affect is considered less desirable in East Asian cultures because it may interfere with fulfilling cultural expectations, such as vigilant attunement to social demands and corresponding adjustment (Tsai et al., 2007). Such a view contrasts with Western formulations wherein high-arousal positive affect (e.g., excitement) is central to hedonic well-being (Tsai et al., 2006; Uchida and Kitayama, 2009). Thus, autonomy and high-arousal positive affect may not be strong predictors of health, including its improvement across time, in Japan.

Such thinking converges with prior work showing cultural differences in associations between autonomy and positive affect with health. Kitayama et al. (2010) found that personal control, which is conceptually similar to autonomy, was positively related to self-rated health among both Japanese and United States adults, but the strength of the association was weaker in Japan. Similarly, Yoo et al. (2017) found that positive affect predicted healthy lipid profiles in the United States, but not in Japan. Clobert et al. (2019) suggest that cultural moderation of the association between positive affect and health might be particularly strong for high-arousal positive affect. Their findings, in fact, showed that high-arousal positive affect predicted self-reported health more strongly in the United States than in Japan, while low-arousal positive affect even showed a reversed pattern (Clobert et al., 2019).

Another aspect of psychological well-being, self-acceptance, includes awareness of not only personal strengths but also weaknesses, which may parallel ideas of self-criticism emphasized in Japanese Confucianism as routes to self-improvement (Heine et al., 2000). Similarly, self-compassion, which may encompass self-acceptance, is central to Buddhist world-views that are deeply rooted in Japanese culture (Dryden and Still, 2006). Self-compassion has been positively linked to supportive friendships and life satisfaction, and negatively to depression among Japanese respondents (Arimitsu, 2014; Yamaguchi et al., 2014; Taniguchi, 2015), while self-acceptance has been negatively linked with depression (Neff et al., 2008). Implications of any of these associations for physical health has not been previously considered.

Environmental mastery, another aspect of psychological well-being studied in the West, may be part of Japanese well-being, although for somewhat different reasons. Being in charge of one’s situation may be valued in Japan not so much in terms of having individual power to influence outcomes, but because of the aim of minimizing difficulties for others (Kim et al., 2006). Thus, this dimension of well-being might be valued in both contexts, albeit for distinct life objectives.

Guided by the above ideas, the purpose of this study was to investigate links between longitudinal profiles of differing aspects of psychological well-being among Japanese adults with distinct aspects of health, also studied across time. Drawing on previous findings with a United States sample (Ryff et al., 2015), we focused on the contrast between those who showed persistently high vs. persistently low well-being across time. The rationale was that the majority of respondents (both in the United States and in Japan) showed stability in their well-being across time, albeit at different levels – some thus had cumulatively high profiles of well-being across time while others had cumulatively low profiles of well-being across time.

The indicators of well-being encompassed six dimensions of eudaimonic well-being (i.e., purpose in life, personal growth, positive relations with others^2^, autonomy, self-acceptance, environmental mastery), and two dimensions of hedonic wellbeing (i.e., high-arousal positive affect, and general/low-arousal positive affect). Four separate measures of self-reported physical health were included: subjective health, chronic conditions, physical health symptoms, and functional health. The objective was to examine whether distinct dimensions of well-being mattered for all, or only some, aspects of cross-time health, thereby offering a comprehensive understanding of how persistently high versus persistently low well-being matters for unfolding profiles of physical health in Japanese adults.

Two overarching predictions guided the inquiry. First, we expected that persistently high levels of psychological well-being in certain domains (i.e., purpose in life, personal growth, positive relations with others) would be associated with health improvement across time in Japan. This prediction draws from prior literature suggesting cultural similarities in these areas of well-being, along with earlier evidence on their associations with health in Japan. Second, we hypothesized that persistently high autonomy and high-arousal positive affect would show weaker links to cross-time health, thus reflecting their reduced salience in the Japanese cultural context. Finally, two other aspects of well-being (self-acceptance, environmental mastery) did not include specific predictions, given the lack of theoretical or empirical guidelines from prior work.

## Materials and Methods

### Participants

Participants were from a large longitudinal survey Midlife in Japan (MIDJA), conducted in 2008 and 2012. The first wave (MIDJA 1; 2008–2009) sampled 1,027 randomly selected adults residing in Tokyo metropolitan areas, aged 30–80. The second wave (MIDJA 2) was conducted in 2012–2013. Of the original 1,027 participants, 657 participants (70%) completed the longitudinal follow-up and are included in the following analyses. Details of the study protocol and data files are available on https://doi.org/10.3886/ICPSR30822.v3.

### Measures

Eudaimonic well-being (Ryff, 1989) included six scales: autonomy, environmental mastery, personal growth, positive relations with others, purpose in life, and self-acceptance. Each scale consisted of seven items rated according to participants’ degree of agreement with each item on a 7-point scale, ranging from 1 (strongly disagree) to 7 (strongly agree). Negative items were reverse-coded such that higher scores indicate higher levels of well-being. The internal consistencies (α) ranged from 0.68 to 0.79.

Hedonic well-being was measured by two types of positive affect. General/low-arousal positive affect was measured by Mroczek and Kolarz’s (1998) scale. Participants were asked to report how frequently they felt each emotion during the past 30 days using of a 5 point-scale (1 = none of the time, 2 = a little of the time, 3 = some of the time, 4 = most of the time, and 5 = all the time). The items of emotions were cheerful, in good spirits, extremely happy, calm, and peaceful, satisfied, full of life. Second, high-arousal positive affect was measured by selective items form PANAS scale (Watson et al., 1988): enthusiastic, proud, active, and attentive. Participants reported the frequency of each item within the past 30 days on the 5-point scale. Alphas ranged from 0.79 to 0.93.

We used four measures of physical health: (1) subjective health, measured by one item asking participants to rate their current health on a scale, ranging from 0 (worst) to 10 (best); (2) number of chronic conditions experienced in the past 12 months (e.g., hypertension, stomach problems, arthritis). The score was constructed by taking the total number of “Yes” responses (ranging from 0 to 30). (3) Frequency of health symptoms measured by aggregating the frequency of nine different symptoms (e.g., headaches, joint stiffness) for the past 30 days (0 = not at all; 1 = once a month, 2 = several times a month, 3 = once a week, 4 = several times a week, and 5 = almost every day; ranging from 0 to 45) The alphas were 0.7 (Time 1) and 0.72 (Time 2). (4) Instrumental activities of daily living scale (IADL) measured functional health. Participants rated the extent to which their health limited various activities of daily living, ranging from basic activities (e.g., lifting or carrying groceries) to vigorous activities (e.g., running) on a 4-point scale (1 = not at all; 2 = a little; 3 = some; 4 = a lot). Thus, higher scores indicated more functional limitations. The alphas were 0.89 for both time points.

Demographic variables included age, gender (0 = male, 1 = female), and level of education (ranging from 1 = no school/some grade school to 8 = Ph.D., M.D., or other professional degree) were included. Table 1 displays descriptive statistics and cross-time correlations for all variables. Measures of psychological well-being were highly correlated over 4–5 years (the average cross-time correlation = 0.67). The health measures showed lower cross-time correlations than well-being variables, with the exception of health symptoms.

**TABLE 1:** Descriptive statistics of key variables at Time 1 and Time 2 (on cases for which longitudinal data were available).

	**Mean (SD)**		**Cross-time *r***
	**Time1**	**Time2**	
Age	54.92 (13.58)	59.25 (13.54)	0.99
Education	4.54 (2.06)	4.55 (2.06)	0.96
% Male	47%	47%	
Autonomy	30.68 (5.5)	30.77 (5.14)	0.73
Environmental mastery	32.02 (5.53)	31.87 (5.19)	0.64
Personal growth	34.03 (5.69)	33.37 (5.67)	0.69
Positive relations	33.71 (5.82)	33.54 (5.57)	0.68
Purpose in life	31.82 (5.16)	31.4 (4.85)	0.64
Self-acceptance	31.13 (5.8)	30.9 (5.39)	0.73
General/low-arousal PA	3.28 (0.77)	3.26 (0.65)	0.64
High-arousal PA	3.09 (0.74)	3.06 (0.77)	0.57
Subjective health	6.36 (1.93)	6.23 (2.05)	0.51
Chronic conditions	2.28 (2.01)	2.13 (1.88)	0.57
Health symptoms	12.02 (7.51)	13.49 (7.91)	0.68
Function health (IADL)	1.39 (0.65)	1.51 (0.76)	0.42

### Longitudinal Profiles of Well-Being

**FIGURE 1:**
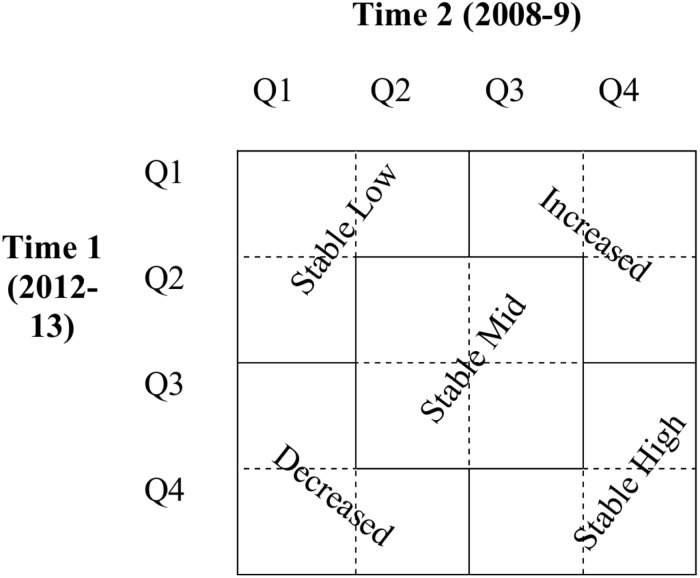
Categorization of longitudinal profiles of well-being based on the quartiles of Time 1 and Time 2 well-being levels.

Consistent with findings from the United States (Ryff et al., 2015), most MIDJA participants showed stable levels of well-being across the 4–5-year period. That is, only 16% or less of the sample reliably changed (Jacobson and Truax, 1991) on any dimension of well-being between the two waves. However, participants were stable at different levels of well-being. To classify individuals’ cumulative well-being over the 4–5 years, respondents were categorized into five groups: persistently low (stable low), persistently moderate (stable mid), persistently high (stable high), increasing and decreasing (see Figure 1). Quartiles cuts at each wave were used to define these groups. Similar to the reliable change index (Jacobson and Truax, 1991), change was defined as moving upward (increasing) or downward (decreasing) 2+ quartiles from Time 1 to Time 2. All other groups were defined as stable, albeit at different levels (low, moderate, high). Table 2 presents the percentages of categories for each well-being dimension. Most Japanese respondents (84–91%) showed high stability over time across all dimensions of well-being. These were apportioned into three varieties of stability: persistently low (24–31%), persistently moderate (29–38%), and persistently high (23–30%). The rest (9–16%) met criteria for increasing or decreasing on the various aspects of well-being.

**TABLE 2:** Categories of longitudinal profiles of well-being.

	**Stable**	**Stable**	**Stable**	**Increase**	**Decrease**
	**low (%)**	**mid (%)**	**high (%)**	**(%)**	**(%)**
**Eudaimonic well-being**					
Autonomy	28.5	34.1	26.6	5.1	5.7
Environmental mastery	27.3	30.7	26.9	8.4	6.7
Personal growth	26.6	35.4	28	6.2	3.7
Positive relations	24.2	34	29.8	6.5	5.4
Purpose in life	30	28.6	25.9	9	6.5
Self-acceptance	23.9	38.2	28.4	5.6	3.9
**Hedonic well-being**					
General	25.9	32.8	29.1	6.5	5.7
High-arousal	31.5	30.7	23.1	8.2	6.5

## RESULTS

The core aim was to test whether persistently high versus low levels of psychological well-being would predict better physical health over 4–5 years. We conducted hierarchical linear regression analyses in which each measure of well-being predicted each measure of health outcome in a separate model. In the first step of the model, demographic variables and Time 1 baseline health were entered to predict Time 2 health. By including Time 1 health, the coefficients represent the associations between predictors and residualized changes in health from Time 1 to Time 2. In the next step, dummy-coded well-being was entered, in which stable high was used as a reference group compared to four other groups (i.e., stable low, stable mid, decreasing, increasing).

The summary of results is displayed in Table 3. Contrasts between stable high and increasing or decreasing groups are not presented because they had few observations, thereby yielding less reliable estimates for the contrasts. Although primary interest was in the contrast between stable high and stable low, the contrast between stable high and stable mid informs the strength of the effect linking different levels of stability in well-being to cross-time changes in health.

**TABLE 3:** The effect sizes (*R*^2^Δ) and unstandardized coefficients (b) of models in which longitudinal well-being predict health outcomes.

**Health measures**		**Subjective health**		**Chronic conditions**		**Health symptoms**		**Functional health**	
		**Stable high vs. stable low**	**Stable high vs. stable mid**	**Stable high vs. stable low**	**Stable high vs. stable mid**	**Stable high vs. stable low**	**Stable high vs. stable mid**	**Stable high vs. stable low**	**Stable high vs. stable mid**
**Eudaimonic well-being**									
Autonomy	*b*	−0.431^∗^	–0.228	0.589^∗∗∗^	0.196	1.684^∗^	1.251^∗^	2.35^∗∗^	2.258^∗∗^
	*R*^2^Δ	0.008		0.017		0.012		0.012	
Environmental mastery	*b*	–0.855^∗∗∗^	−0.377^∗^	0.875^∗∗∗^	0.184	2.609^∗∗∗^	1.111^+^	2.904^∗∗^	2.512^∗∗^
	*R*^2^Δ	0.034		0.033		0.017		0.015	
Personal growth	*b*	–0.806^∗∗∗^	−0.349^∗^	0.516^∗∗^	0.266^+^	1.22^+^	1.003	2.493^∗∗^	1.103
	*R*^2^Δ	0.031		0.010		0.01		0.009	
Positive relations	*b*	–0.950^∗∗∗^	–0.476^∗∗^	0.314^+^	0.056	1.705^∗^	1.754^∗∗^	2.552^∗∗^	0.029
	*R*^2^Δ	0.037		0.008		0.015		0.013	
Purpose in life	*b*	–0.617^∗∗^	–0.280	0.492^∗∗^	0.038	1.82^∗∗^	1.081	2.883^∗∗^	1.366
	*R*^2^Δ	0.018		0.018		0.007		0.014	
Self-acceptance	*b*	–0.797^∗∗∗^	–0.759^∗∗∗^	0.586^∗∗^	0.083	1.689^∗^	0.038	2.904^∗∗^	1.963^∗^
	*R*^2^Δ	0.042		0.014		0.008		0.013	
**Hedonic well-being**									
General/low-arousal	*b*	–0.906^∗∗∗^	–0.460^∗∗^	0.702^∗∗∗^	0.300^+^	0.945	0.712	1.237	0.704
	*R*^2^Δ	0.032		0.023		0.011		0.003	
High-arousal	*b*	–0.745^∗∗∗^	–0.234	0.492^∗∗^	0.160	1.314^+^	0.759	2.317^∗∗^	1.774^∗^
	*R*^2^Δ	0.028		0.010		0.007		0.010	

*Models included age, gender, education, and Time 1 health as covariates. ^∗∗∗^*p* < 0.001, ^∗∗^*p* < 0.01, ^∗^*p* < 0.05, ^+^*p* < 0.1.*

### Subjective Health

For all six scales of eudaimonic well-being, respondents with stable low profiles had worse subjective health at Time 2 compared to those with stable high profiles. For environmental mastery, personal growth, and positive relations with others, those with stable mid well-being also had worse health compared to those with stable high well-being. Autonomy explained smaller variances, *R*^2^Δ = 0.008, compared to other dimensions of eudaimonic well-being, *R*^2^Δ = 0.018–0.042.

For both measures of hedonic well-being, those with stable low profiles showed worse health than those with stable high profiles. In addition, for general/low-arousal positive affect, those with stable mid profiles had worse health than those with stable high profiles. The effect size of general/low-arousal positive affect was slightly larger, *R*^2^Δ = 0.032, than high-arousal positive affect, *R*^2^Δ = 0.028.

### Chronic Conditions

For all scales of eudaimonic well-being except for positive relations with others, those with stable low well-being reported more chronic conditions in 5 years compared to those with stable high well-being. Unlike subjective health, the effect size of autonomy was, *R*^2^Δ = 0.017, comparable with other dimensions (e.g., self-acceptance: *R*^2^Δ = 0.014).

For both measures of hedonic well-being, those with stable low profiles reported greater increments in chronic conditions than those with stable high profiles. General/low-arousal positive affect explained more variance of the chronic conditions, *R*^2^Δ = 0.023, compared to high-arousal positive affect, *R*^2^Δ = 0.010.

### Health Symptoms

The increase in health symptoms was greater for those with stable low well-being than those with stable high well-being, across all dimensions of eudaimonic well-being except personal growth. In additions, those with stable mid-levels of positive relations with others and autonomy had more health symptoms compared to those with the stable high-levels. Consistent with the results for chronic conditions, the effect size of autonomy was comparable to other dimensions of eudaimonic well-being. For hedonic well-being, neither general/low-arousal nor high-arousal positive affect predicted changes in health symptoms.

### Functional Health

Those with stable low well-being reported more physical limitations in daily activities than those with stable high well-being across all dimensions of eudaimonic well-being. For environmental mastery, autonomy, and self-acceptance, those with stable mid well-being had worse functional health compared to those with stable high well-being. Again, the effect size of autonomy was comparable to other dimensions of eudaimonic well-being.

The association between hedonic well-being and functional health for daily activities showed a different pattern from other health outcomes. Cumulative profiles of high-arousal positive affect predicted greater increments in functional health whereas the profiles of general/low-arousal positive affect did not predict functional health. Those with stable high high-arousal positive affect reported relative improvement in functional health compared to those with stable low and stable mid high-arousal positive affect.

## Discussion

The study yielded consistent patterns of results across dynamic measures of health that were predicted by cumulative profiles of diverse aspects of psychological well-being in Japanese adults. Specifically, persistently high levels of psychological well-being predicted improved subjective health, fewer chronic conditions, fewer health symptoms, and fewer functional health problems over 4–5 years compared to persistently low levels of psychological well-being. Persistently high levels of psychological well-being also predicted better subjective health, fewer health symptoms, and fewer functional limitations compared to persistently moderate levels of psychological well-being, suggesting that health improvement associated with higher psychological well-being may not be limited to those with extreme levels of psychological well-being. Together, the findings extend a prior literature focused on longitudinal associations between psychological well-being and health in United States adults to another cultural context, namely, Japan.

For all six dimensions of eudaimonic well-being, Japanese adults with persistently high profiles of well-being showed greater health improvement (i.e., higher subjective healthy, reduced chronic conditions and health symptoms, fewer functional limitations) than those with stable low profiles of well-being. This was true even for autonomy, which is less culturally valued in in Japan compared to the United States It is notable that such effects were evident despite a notably shorter follow-up period in Japan (4–5 years) compared to the longer period (9–10 years) found for United States adults (Ryff et al., 2015). Overall, the patterns convey that cumulatively high levels of eudaimonic well-being point to similarly benefits for cross-time health in both Japan and the United States.

For hedonic well-being, persistently high levels of both low- and high-arousal positive affect predicted improved subjective health and fewer chronic conditions than persistently low levels of hedonic well-being. The results partially supported our hypotheses that high-arousal positive affect would show weaker association with health than general/low-arousal positive affect in Japan, except for functional health. These are consistent with prior research showing that low-arousal positive affect was more strongly associated with health than high-arousal positive affect in Japan (Clobert et al., 2019). Cumulative profiles of hedonic well-being did not predict change in health symptoms, contrary to eudaimonic well-being. Given that health symptoms were the most stable measure of health over 4–5 years (cross-time *r* = 0.68), it can be speculated that the effect of hedonic well-being on change in health may be more contingent on the stability of health measures than eudaimonic well-being.

Lastly, change in functional health was associated with only high-arousal positive affect. The different results for functional health could be attributed to that its measure included engagement in high-arousal activities (i.e., running). To investigate this possibility, we ran three separate models testing whether high-arousal positive affect measured at Time 1, Time 2, or the change between the two time points would predict Time 2 functional health (controlling for Time 1 functional health). The only significant predictor was Time 2 high-arousal positive affect, indicating that the findings are accounted by the concurrent association between high-arousal positive affect and functional health, rather than the effect of high-arousal positive affect on health over time.

Overall, psychological well-being accounted for small variances in Time 2 health. These small effect sizes were expected because the models predicted Time 2 health adjusted for Time 1 health; the residual changes in health were small between the relatively short interval (i.e., 4–5 years). Nonetheless, the small effect sizes were comparable to prior studies with Western samples that examined the associations between psychological well-being and prospective health outcomes such as mortality and longevity (Chida and Steptoe, 2008; Xu and Roberts, 2010), suggesting that even small effects can have practical implications.

The key limitation of the study is that the analyses cannot rule out the reversed causality between well-being and health. Cumulative profiles of well-being were created based on Time 2 as well as baseline measure, which do not temporally precede Time 2 health. Thus, the associations between health and persistent levels of well-being could be partly due to the effect of health improvement on well-being. With the exception of the result for functional health and high arousal positive affect, however, the variance of Time 2 health (net of Time 1 health) explained by cross-time well-being was greater than the variance of Time 2 well-being (net of Time 1 well-being) explained by cross-time health. This suggests that the observed associations were likely derived by the influence of cross-time well-being on health, though the alternative explanation cannot be rejected.

In addition, the findings are based on single-sourced, self-reported measures of health. Although prior studies have shown that self-reported health including subjective health and chronic conditions are valid indicators of physical health (e.g., Bound, 1989; Idler and Benyamini, 1997; Miilunpalo et al., 1997), future research should examine whether the effects can be expanded to objective measures of health from multiple sources (e.g., Radler et al., 2018). Moreover, the current study used longitudinal data only measured at two time points, which did not allow other statistical methods that classify longitudinal profiles of continuous variables with more precision (e.g., latent transition analysis). Nonetheless, the current approach of focusing on cumulative profiles of well-being aimed to provide a starting point to investigate effects of stable levels of well-being on dynamic aspects of health.

Although comprehensive measures of well-being were employed, all were developed based on the theories formulated in the Western cultures. It is possible that these measures miss certain aspects of well-being specific to Japanese cultures. For instance, the study did not have an adequate measure of harmonious social relationships that receives a great emphasis in Japanese cultures. Although positive relations with others was included as an aspect of well-being, this measure focuses on perceptions of warm and trusting relationships (Ryff, 1989) whereas harmonious social relationships in Japan are often evaluated at a group level, including external viewpoints (e.g., fulfilling expectations of close others). Including more culturally relevant measures of well-being might reveal even stronger effect sizes in associations between well-being with health in Japan.

Despite the limitations, the study is unique in that it examined multiple dimensions of psychological well-being to test longitudinal relationships between cumulative well-being and change in health in a different culture. The use of multi-dimensional approach is particularly important in considering cultural contexts, given that some dimensions have more cultural variation than others. Furthermore, by focusing on the stable levels of well-being, the study was able to examine health implications of well-being for which most people do not show reliable changes. The findings showed health predictability of persistent level of psychological well-being even when controlling for baseline health in Japan, highlighting the importance of cumulative psychological well-being in health benefits in diverse cultural contexts.

## Data Availability Statement

The datasets generated for this study are available on request to the corresponding author.

## Ethics Statement

The studies involving human participants were reviewed and approved by the University of Wisconsin–Madison Institutional Review Board. The patients/participants provided their written informed consent to participate in this study.

## Author Contributions

JY carried out the data analysis and drafted the manuscript under the supervision of CR. Both authors developed the study concept and approved the final version of the manuscript for submission.

## Conflict of Interest

The authors declare that the research was conducted in the absence of any commercial or financial relationships that could be construed as a potential conflict of interest.

## Footnotes

1 In Western cultures, positive relations with others has been consistently conceptualized as a central part of well-being (Ryff, 1989; Diener and Seligman, 2002), and have been linked to better health (Cohen, 2004), underscoring that individualism of Western cultures is not at the expense of social relations. Positive social relations fulfill universal human need to affiliate (Deci and Ryan, 2000) although cultural differences may exist in the specific nature of social relations.

2 Although a certain aspect of social relations (i.e., maintaining social harmony) may have a greater importance in interdependent cultures than in independent cultures, the current study employed the conceptualization and measurement of social well-being established in Western cultures. Thus, we did not expect positive social relations to show a stronger association with health comparing to purpose in life or personal growth.

## References

[B1] Arimitsu K. (2014). Development and validation of the Japanese version of the self-compassion scale. *Jap. J. Psychol.* 85 50–59. 10.4992/jjpsy.85.50 24804430

[B2] Benyamini Y. Idler E. L. Leventhal H. Leventhal E. A. (2000). Positive affect and function as influences on self-assessments of healthexpanding our view beyond illness and disability. *J. Gerontol. B* 55 107–116. 10.1093/geronb/55.2.P107 10794189

[B3] Bound J. (1989). *Self-Reported vs. Objective Measures of Health in Retirement Models. NBER Working Papers 2997.* Cambridge, MA: National Bureau of Economic Research.

[B4] Boyle P. A. Barnes L. L. Buchman A. S. Bennett D. A. (2009). Purpose in life is associated with mortality among community-dwelling older persons. *Psychosom. Med.* 71:574. 10.1097/PSY.0b013e3181a5a7c0 19414613 PMC2740716

[B5] Chida Y. Steptoe A. (2008). Positive psychological well-being and mortality: a quantitative review of prospective observational studies. *Psychosom. Med.* 70 741–756. 10.1097/PSY.0b013e31818105ba 18725425

[B6] Clobert M. Sims T. L. Yoo J. Miyamoto Y. Markus H. R. Karasawa M. (2019). Feeling excited or taking a bath: do distinct pathways underlie the positive affect–health link in the U.S. and Japan. *Emotion* 10.1037/emo0000531 [Epub ahead of print]. 30676038 PMC6656630

[B7] Cohen S. (2004). Social relationships and health. *Am. Psychol.* 59 676–684. 10.1037/0003-066X.59.8.676 15554821

[B8] Deci E. L. Ryan R. M. (2000). The ‘What’ and ‘Why’ of goal pursuits: human needs and the self-determination of behavior. *Psychol. Inq.* 11 227–268.

[B9] Diener E. Chan M. Y. (2011). Happy people live longer: subjective well-being contributes to health and longevity. *Appl. Psychol. Health Well Being* 3 1–43. 10.1111/j.1758-0854.2010.01045.x

[B10] Diener E. Seligman M. E. P. (2002). Very happy people. *Psychol. Sci.* 13 81–84. 11894851 10.1111/1467-9280.00415

[B11] Dryden W. Still A. (2006). Historical aspects of mindfulness and self-acceptance in psychotherapy. *J. Ration. Emot. Cogn. Behav. Ther.* 24 3–28. 10.1007/s10942-006-0026-1

[B12] Friedman E. M. Ryff C. D. (2012). Living well with medical comorbidities: a biopsychosocial perspective. *J. Gerontol. B* 67 535–544. 10.1093/geronb/gbr152 22377799 PMC3441187

[B13] Fulmer C. A. Gelfand M. J. Kruglanski A. W. Kim-Prieto C. Diener E. Pierro A. (2010). On ‘feeling right’ in cultural contexts: how person-culture match affects self-esteem and subjective well-being. *Psychol. Sci.* 21 1563–1569. 10.1177/0956797610384742 20876880

[B14] Heine S. J. Takata T. Lehman D. R. (2000). Beyond self-presentation: evidence for self-criticism among Japanese. *Pers. Soc. Psychol. Bull.* 26 71–78. 10.1177/0146167200261007

[B15] Hill P. L. Turiano N. A. (2014). Purpose in life as a predictor of mortality across adulthood. *Psychol. Sci.* 25 1482–1486. 10.1177/0956797614531799 24815612 PMC4224996

[B16] Idler E. L. Benyamini Y. (1997). Self-rated health and mortality: a review of twenty-seven community studies. *J. Health Soc. Behav.* 38 21–37. 9097506

[B17] Jacobson N. S. Truax P. (1991). Clinical significance: a statistical approach to defining meaningful change in psychotherapy research. *J. Consult. Clin. Psychol.* 59 12–19. 10.1037/0022-006x.59.1.12 2002127

[B18] Karasawa M. Curhan K. B. Markus H. R. Kitayama S. S. Love G. D. Radler B. T. (2011). Cultural perspectives on aging and well-being: a comparison of Japan and the United States. *Int. J. Aging Hum. Dev.* 73 73–98. 10.2190/AG.73.1.d 21922800 PMC3183740

[B19] Kim E. S. Sun J. K. Park N. Peterson C. (2013). Purpose in life and reduced incidence of stroke in older adults:’the health and retirement study’. *J. Psychosom. Res.* 74 427–432. 10.1016/j.jpsychores.2013.01.013 23597331

[B20] Kim H. S. Sherman D. K. Ko D. Taylor S. E. (2006). Pursuit of comfort and pursuit of harmony: culture, relationships, and social support seeking. *Pers. Soc. Psychol. Bull.* 32 1595–1607. 10.1177/0146167206291991 17122173

[B21] Kitayama S. Karasawa M. Curhan K. B. Ryff C. D. Markus H. R. (2010). Independence and interdependence predict health and wellbeing: divergent patterns in the United States and Japan. *Front. Psychol.* 1:163. 10.3389/fpsyg.2010.00163 21833228 PMC3153777

[B22] Kitayama S. Markus H. R. (2000). “The pursuit of happiness and the realization of sympathy,” in *Cultural Patterns of Self, Social Relations, and Well-Being. Culture and Subjective Well-Being*, eds Diener Ed. Suh E. M. (Cambridge, MA: The MIT Press)

[B23] Kitayama S. Markus H. R. Kurokawa M. (2000). Culture, emotion, and well-being: good feelings in Japan and the United States. *Cogn. Emot.* 14 93–124. 10.1080/026999300379003

[B24] Kitayama S. Park H. (2007). Cultural shaping of self, emotion, and well-being: how does it work? *Soc. Pers. Psychol. Comp.* 1 202–222. 10.1111/j.1751-9004.2007.00016.x

[B25] Kumano M. (2017). On the concept of well-being in Japan: feeling shiawase as hedonic well-being and feeling ikigai as eudaimonic well-being. *Appl. Res. Qual. Life* 13 419–433. 10.1007/s11482-017-9532-9

[B26] Markus H. R. Kitayama S. (1991). Culture and the self: implications for cognition, emotion, and motivation. *Psychol. Rev.* 98 224–253. 10.1037//0033-295x.98.2.224

[B27] Miilunpalo S. Vuori I. Oja P. Pasanen M. Urponen H. (1997). Self-rated health status as a health measure: the predictive value of self-reported health status on the use of physician services and on mortality in the working-age population. *J. Clin. Epidemiol.* 50 517–528. 10.1016/S0895-4356(97)00045-0 9180644

[B28] Miyamoto Y. Yoo J. Wilken B. (2019). “Well-being and health: a cultural psychology of optimal human functioning,” in *Handbook of Cultural Psychology*, eds Cohen D. Kitayama S. (New York, NY: Guilford).

[B29] Mroczek D. K. Kolarz C. M. (1998). The effect of age on positive and negative affect: a developmental perspective on happiness. *J. Pers. Soc. Psychol.* 75 1333–1349. 10.1037/0022-3514.75.5.1333 9866191

[B30] Nakanishi N. (1999). ’Ikigai’in older Japanese people. *Age Ageing* 28 323–324. 10.1093/ageing/28.3.323 10475874

[B31] Neff K. D. Pisitsungkagarn K. Hsieh Y.-P. (2008). Self-compassion and self-construal in the United States, Thailand, and Taiwan. *J. Cross Cult. Psychol.* 39 267–285. 10.1177/0022022108314544

[B32] Oishi S. (2000). *Goals as Cornerstones of Subjective Well-Being: Linking Individuals and Cultures. in: Culture and Subjective Well-Being.* Cambridge, MA: The MIT Press, 87–112.

[B33] Radler B. T. Rigotti A. Ryff C. D. (2018). Persistently high psychological well-being predicts better HDL cholesterol and triglyceride levels: findings from the midlife in the U.S. (MIDUS) longitudinal study. *Lipids Health Dis.* 17:1. 10.1186/s12944-017-0646-8 29298716 PMC5751819

[B34] Ryff C. D. (1989). Happiness is everything, or is it? explorations on the meaning of psychological well-being. *J. Pers. Soc. Psychol.* 57 1069–1081. 10.1037/0022-3514.57.6.1069 26400043

[B35] Ryff C. D. Love G. D. Miyamoto Y. Markus H. R. Curhan K. B. Kitayama S. (2014). “Culture and the promotion of well-being in east and west: understanding varieties of attunement to the surrounding context,” In *Increasing Psychological Well-Being in Clinical and Educational Settings. Cross-Cultural Advancements in Positive Psychology*, eds Fava G. Ruini C. (Dordrecht: Springer), 1–19 10.1007/978-94-017-8669-0_1

[B36] Ryff C. D. Radler B. T. Friedman E. M. (2015). Persistent psychological well-being predicts improved self-rated health over 9–10 years: longitudinal evidence from MIDUS. *Health Psychol. Open* 2:205510291560158. 10.1177/2055102915601582 26617988 PMC4662422

[B37] Sone T. Nakaya N. Ohmori K. Shimazu T. Higashiguchi M. Kakizaki M. (2008). Sense of life worth living (ikigai) and mortality in Japan: Ohsaki study. *Psychosom. Med.* 70 709–715. 10.1097/PSY.0b013e31817e7e64 18596247

[B38] Taniguchi H. (2015). Interpersonal mattering in friendship as a predictor of happiness in Japan: the case of tokyoites. *J. Happiness Stud.* 16 1475–1491. 10.1007/s10902-014-9570-z

[B39] Tanno K. Sakata K. Ohsawa M. Onoda T. Itai K. Yaegashi Y. (2009). Associations of ikigai as a positive psychological factor with all-cause mortality and cause-specific mortality among middle-aged and elderly Japanese people: findings from the Japan collaborative cohort study. *J. Psychosom. Res.* 67 67–75. 10.1016/j.jpsychores.2008.10.018 19539820

[B40] Tsai J. L. (2017). Ideal affect in daily life: implications for affective experience, health, and social behavior. *Curr. Opin. Psychol.* 17 118–128. 10.1016/j.copsyc.2017.07.004 28950957 PMC5659332

[B41] Tsai J. L. Knutson B. Fung H. H. (2006). Cultural variation in affect valuation. *J. Pers. Soc. Psychol.* 90 288–307. 10.1037/0022-3514.90.2.288 16536652

[B42] Tsai J. L. Miao F. F. Seppala E. Fung H. H. Yeung D. Y. (2007). Influence and adjustment goals: sources of cultural differences in ideal affect. *J. Pers. Soc. Psychol.* 92 1102–1117. 10.1037/0022-3514.92.6.1102 17547491

[B43] Uchida Y. Kitayama S. (2009). Happiness and unhappiness in east and west: themes and variations. *Emotion* 9 441–456. 10.1037/a0015634 19653765

[B44] Ueshima K. Fujiwara T. Takao S. Suzuki E. Iwase T. doi H. (2010). Does social capital promote physical activity? a population-based study in Japan. *PLoS One* 5:e12135. 10.1371/journal.pone.0012135 20808822 PMC2924608

[B45] Watson D. Clark L. A. Tellegen A. (1988). Development and validation of brief measures of positive and negative affect: the PANAS scales. *J. Pers. Soc. Psychol.* 54 1063–1070. 10.1037//0022-3514.54.6.1063 3397865

[B46] Xu J. Roberts R. E. (2010). The power of positive emotions: it’s a matter of life or death–subjective well-being and longevity over 28 years in a general population. *Health Psychol.* 29 9–9. 10.1037/a0016767 20063931

[B47] Yamaguchi A. Kim M.-S. Akutsu S. (2014). The effects of self-construals, self-criticism, and self-compassion on depressive symptoms. *Pers. Individ. Diff.* 68 65–70. 10.1016/j.paid.2014.03.013

[B48] Yoo J. Miyamoto Y. Rigotti A. Ryff C. D. (2017). Linking positive affect to blood lipids: a cultural perspective. *Psychol. Sci.* 28 1468–1477. 10.1177/0956797617713309 28817363 PMC5633496

